# Prophylactic embolization of the Corona mortis in a patient with multiple pelvic fractures: a tool to avoid perioperative bleeding?

**DOI:** 10.1186/s42155-026-00684-w

**Published:** 2026-06-12

**Authors:** Luca Joel De Simone, Andrea Guarino, Fulvio Morrone, Giovanni Barbato, Mauro Nese, Daniele Giuseppe Romano

**Affiliations:** 1 U.O.C Radiologia Interventistica e Vascolare, Dipartimento di Diagnostica per Immagini, A.O.U. San Giovanni Di Dio e Ruggi D’Aragona, Salerno, Italy; 2 U.O.C. Ortotraumatologia, A.O.U. Ospedale San Giovanni Di Dio e Ruggi d’Aragona, Salerno, Italy; 3 U.O.S.D. Radiologia Vascolare ed Interventistica, Ospedale del Mare, Naples, Italy; 4 U.O.C Neuroradiologia diagnostica ed Interventistica, Dipartimento di Diagnostica per Immagini, A.O.U. San Giovanni Di Dio e Ruggi D’Aragona, Salerno, Italy

## Background

**Fig. 1:**
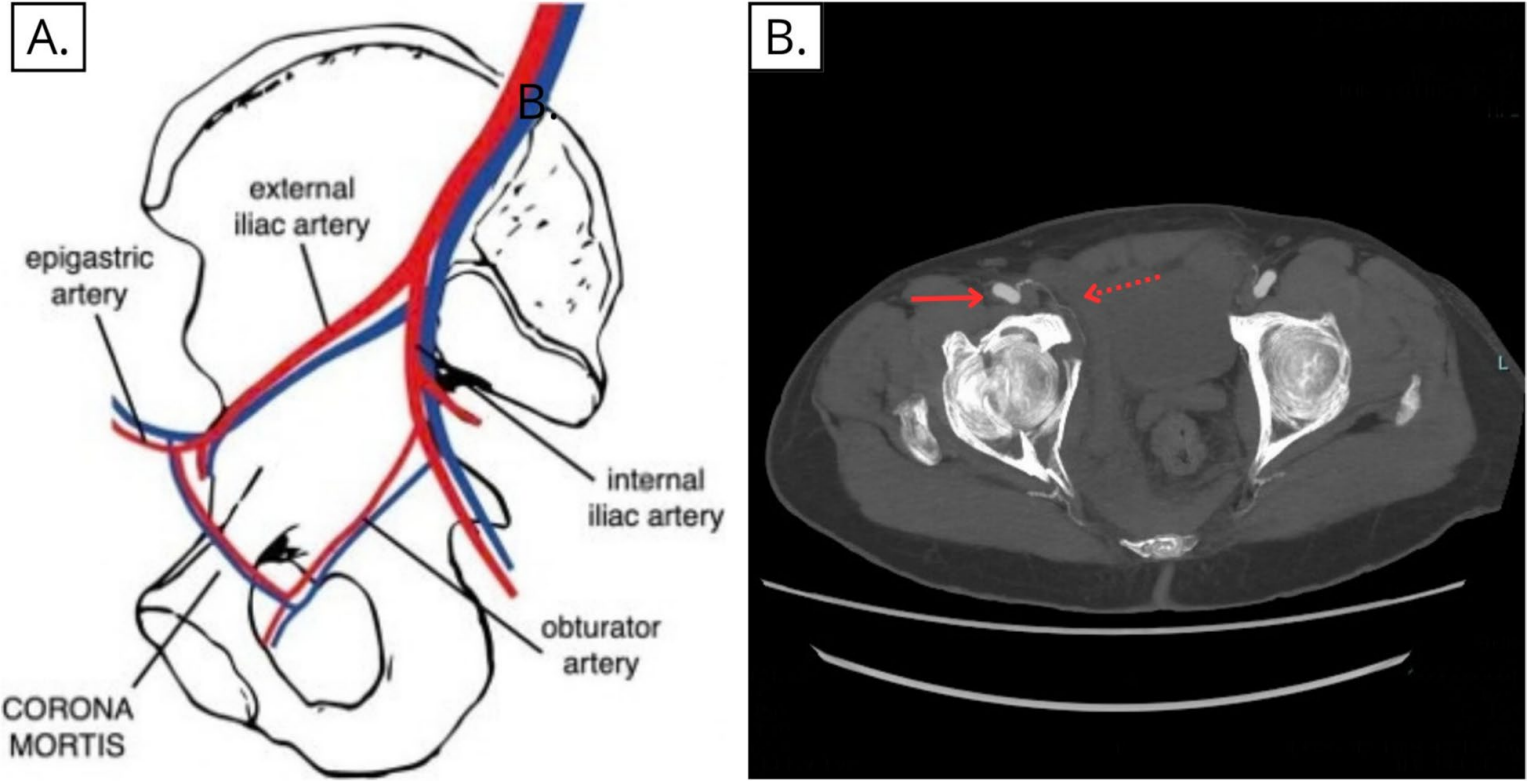
**A** Schematic view of the CM with a medial view on the pelvis (Samuel Friedrich [11]),**B** AngioCT scan showing CM (dashed red arrow) arising from external iliac artery (continuous red arrow)

To the Editor,

Corona mortis (CM) is an anatomical variant characterized by a vessel anastomosis between the external iliac artery and the obturator vessels (Fig. 1A). CM stands for “Crown of Death” in Latin because of an injury in this structure may result in significant bleeding [1]. CM can be arterial and/or venous and can also be classified as bilateral or unilateral. Incidence of CM is 63% in both arterial and venous variants and the mean caliber is calculated to be 2.8 mm, with a more unilateral incidence and no specific side incidence [2]. Traumatic pelvic injuries involving the anterior region carry an increased risk of CM injury. The most commonly associated fracture types include anterior column and anterior wall acetabular fractures, superior pubic ramus fractures, and Young-Burgess Anterior–Posterior Compression grade 2–3 as well as lateral compression grade 2–3 fractures [3]. The most common iatrogenic damage occurs in surgical and orthopedic interventions, such as inguinal or femoral hernia repairs and hip fractures [4] or gynecological interventions [5]. Several surgical approaches such as the modified Stoppa approach [6], the pararectus approach [7], and the ilioinguinal approach [8] carry a risk of injury of the CM [9]. Therefore, careful identification and protection of the CM is required to prevent intraoperative bleeding complications [10].

## Case presentation

**Fig. 2:**
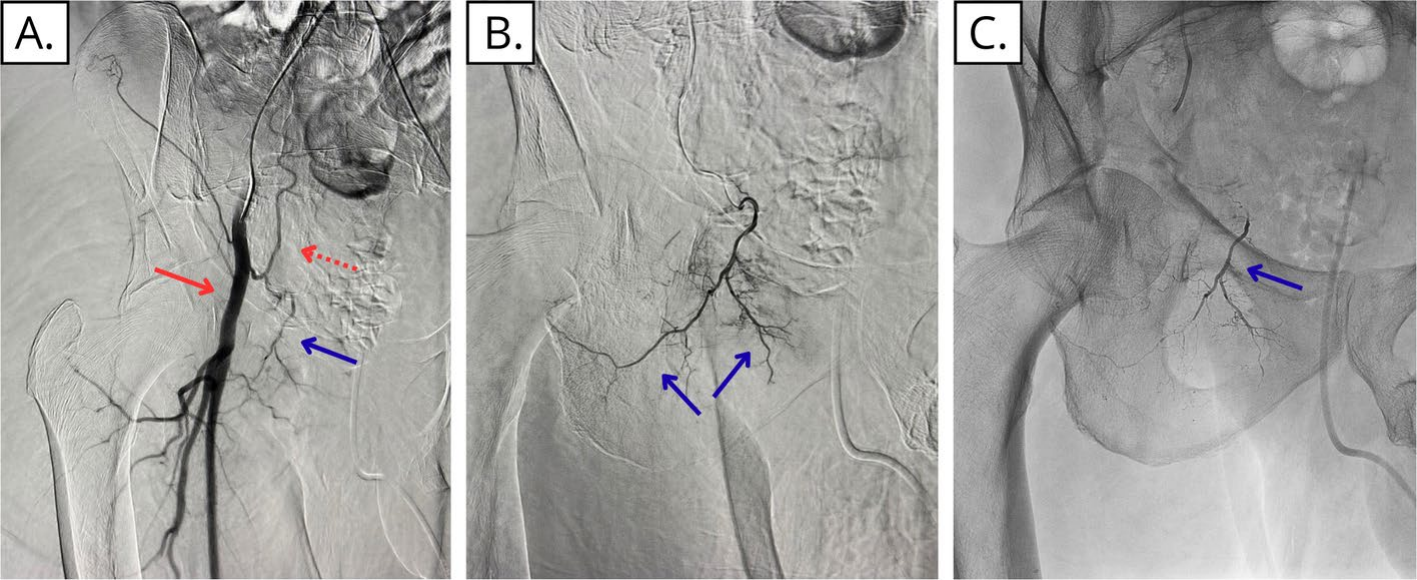
**A** Pre-embolization digital subtraction angiography (DSA) of CM (continuous blue arrow) and inferior epigastric artery (dashed red arrow) performed from external iliac artery (continuous red arrow); **B** Superselective catheterization with microcatheter of CM. DSA shows anastomoses with obturator artery (continuous blue arrows); **C** Post-embolization angiography of CM (continuous blue arrow)

A 72-year-old man with no significant underlying health conditions presented to the emergency department following a traumatic event not in life-threatening danger. A CT scan revealed a multifragmentary fracture involving the right acetabulum with displacement of the femoral head, which was additionally fractured in its superoanterior portion. Fractures of the iliopubic and ischiopubic branches were also identified on the same side. Subsequent CT angiography demonstrated no active bleeding, despite the presence of an extraperitoneal hematoma localized to the perivesical area, both iliac fossae and the presacral region. Despite significant blood loss, blood transfusions were not required as the patient did not suffer from severe anemia (Hb 11.6 g/dL on admission to the emergency room). The imaging revealed the presence of a right-sided CM (Fig. 1B), characterized by an anastomotic branch originating from the proximal segment of the inferior epigastric artery and connecting with the ipsilateral obturator artery. This vessel measured approximately 1.9 mm in diameter. Based on these findings, the orthopedic surgeon requested preoperative embolization of the anastomotic branch to reduce the risk of intraoperative bleeding. Angiographic imaging from the right external iliac artery (LAO 26°, CAU 3°) revealed a common trunk giving rise to the inferior epigastric artery and the CM (Fig. 2A). The CM was superselectively catheterized using an Excelsior SL-10 (Stryker) microcatheter and a Synchro 0.14 (Stryker) microguidewire. Angiographic runs confirmed its connection with the ipsilateral obturator artery (Fig. 2B). Superselective embolization of the CM was then performed using NBCA (Glubran 2—GEM Italy) diluted with Lipiodol (Guerbet) at a ratio of 3:1. Following embolization, DSA confirmed the presence of embolic material along the CM and the exclusion of the anastomosis (Fig. 2C). The same technique was used to embolize the inferior epigastric artery, as requested by the clinical team, in order to minimize the risk of injury to the artery during surgery to stabilize the fractures [12]. Post-procedural non-contrast ConeBeam CT was performed, confirming effective embolization of both vascular branches up to the common trunk emerging from the external iliac artery.

**Fig. 3:**
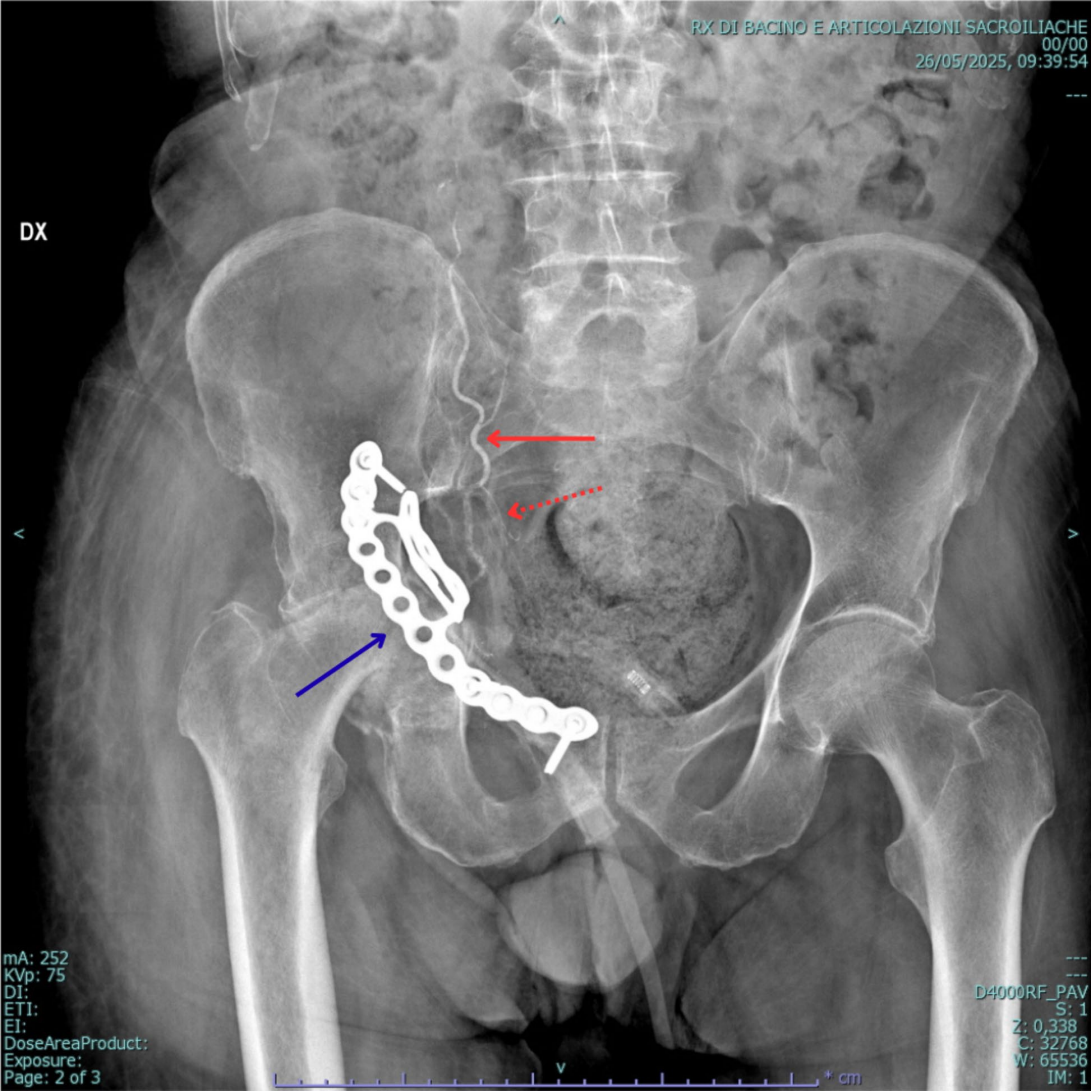
A 24-day X-ray examination of the pelvis showing osteosynthesis devices (continuous blue arrow) and radiopaque embolic material visible within the CM (dashed red arrow) and the inferior epigastric artery (continuous red arrow)

Two days after embolization, the patient underwent orthopedic surgery with the use of osteosynthesis devices. No intraoperative complications were reported and no blood transfusion was required (Hb 9.3 g/dL after the surgery versus Hb 10.3 g/dL pre-surgery). Twelve days after, he was discharged in good general conditions. A 24-day X-ray examination of the pelvis confirms the successful outcome of the orthopedic surgery in the absence of long-term complications related to the embolization procedure (Fig. 3).

## Discussion

A review of the available literature did not reveal procedure-related complications following embolization of the CM. In a retrospective series of 14 patients undergoing transcatheter arterial embolization of the CM for pelvic trauma hemorrhage, there were no reported complications directly attributable to the embolization procedure, including ischemic injury, non-target embolization, or access site complications [13]. Additionally, case reports describing superselective embolization techniques for CM hemorrhage have not documented any adverse events or post-procedural complications (Xinjian [14]). While the theoretical risks of embolization in this vascular territory include pelvic organ ischemia, gluteal or thigh necrosis, or inadvertent embolization of adjacent vessels, these have not been observed in the published cases to date. The risk–benefit ratio of pre-surgical embolization of the CM vessel in patients undergoing orthopedic and gynecological surgeries seems to be highly favorable in selected high-risk cases. The benefits such as reduction in intraoperative blood loss, transfusion requirements, and operative time are well-documented [13], while the risk of major complications is exceedingly low when embolization is performed by experienced interventionalists using superselective techniques. Patient selection should be guided by preoperative imaging, anatomical considerations, and individual comorbidities, as well as the complexity and invasiveness of the planned procedure.

## Data Availability

Data sharing is not applicable to this article as no datasets were generated or analyzed during the current study.
